# Effectiveness of Nutrition and WASH/malaria educational community-based interventions in reducing anemia in children from Angola

**DOI:** 10.1038/s41598-021-85006-x

**Published:** 2021-03-10

**Authors:** Cláudia Fançony, Ânia Soares, João Lavinha, Henrique Barros, Miguel Brito

**Affiliations:** 1 Health Research Center of Angola (CISA, Translated), Caxito, Angola; 2 Instituto de Saúde Pública da Universidade Do Porto, Porto, Portugal; 3 Departamento de Genética Humana, Instituto Nacional de Saúde Dr. Ricardo Jorge, Lisbon, Portugal; 4 BioISI, Faculdade de Ciências, Universidade de Lisboa, Lisbon, Portugal; 5 Health and Technology Research Center (H&TRC), Escola Superior de Tecnologia da Saúde de Lisboa, Instituto Politécnico de Lisboa, Lisbon, Portugal

**Keywords:** Infectious diseases, Risk factors

## Abstract

We found no published data in Angola regarding the effect of combining nutrition-specific and nutrition-sensitive approaches in the reduction of anemia in preschool children. Thus, we implemented a cluster-randomized controlled trial to determine the effectiveness of two educational-plus-therapeutic interventions, in Nutrition and WASH/Malaria, in reducing anemia. We compared them to (1) a test-and-treat intervention and (2) with each other. A block randomization was performed to allocate 6 isolated hamlets to 3 study arms. A difference-in-difference technique, using Fit Generalized estimating models, was used to determine differences between the children successfully followed in all groups, between 2015 and 2016. We found no significant differences in anemia´s and hemoglobin variability between educational and the control group. However, the WASH/Malaria group had 22.8% higher prevalence of anemia when compared with the Nutrition group, having also higher prevalence of *P. falciparum*. Thus, our results suggest that adding a 12-month educational Nutrition or a WASH/Malaria component to a test-and-treat approach may have a limited effect in controlling anemia. Possibly, the intensity and duration of the educational interventions were not sufficient to observe the amount of behavior change needed to stop transmission and improve the general child feeding practices.

## Introduction

In sub-Saharan Africa, controlling infectious morbidity through drug therapy, alongside increasing micronutrient intake, has been the most used approach to interrupt the processes by which infectious diseases cause anemia (such as blood loss, inflammation, hemolysis and blunted intestinal villi) and may increase the impact of health interventions^1–3^. However, controlling schistosomiasis, intestinal parasites and malaria exclusively through drug therapy may have limited sustainability in contaminated environment and/or in settings with inadequate water, sanitation and hygiene and malaria preventive practices and behaviors, because high re-infection rates may occur^4–6^. Thus, an educational component is essential to limit transmission.

A nutrition-sensitive approach could increase hemoglobin based on the assumption that providing health education and promoting adequate Water, Sanitation and Hygiene and malaria (WASH/Malaria) preventive practices would lead to improved knowledge and awareness of mothers/caretakers regarding the causes, prevention and treatment of urogenital schistosomiasis, intestinal parasite and malaria. This, would in turn reduce inadequate practices and risk behaviors (child´s, mother´s and mother-to-child practices) and therefore reducing prevalence, incidence and re-infection rates of those diseases^5–10^.

Nevertheless, nutrition-specific approaches are crucial to address micronutrient deficiencies caused by inadequate Infant and Young Child Feeding Practices (IYCF), which could also benefit from increased sustainability if combined with a therapeutic approach. A nutrition educational intervention could reduce anemia based on the assumption that the intervention would improve the knowledge and awareness of mothers and caretakers regarding adequate feeding practices, which in turn could be translated into behavior changes resulting in improved nutritional quality of the food consumed by the child. This, could finally be reflected in decreased anemia and increased hemoglobin levels^11–15^.

The overall goal of educational interventions is to empower individuals to improve the quality of their own diets, and/or WASH and malaria preventive practices^16–18^. Furthermore, the design of these interventions may vary according to the target population (adult or children), dimensions of the learning package (e.g. for WASH: stool disposal, water quality or supply, for malaria prevention: bednet usage and vector control, and for nutrition: nutritional diversity, consumption of specific micronutrient-rich foods), the delivers (community promoters/volunteers, teachers, local health workers, community leaders), the place where education occurs (domiciliary, at the health center or at community classes), the number of intervention contacts, type of contacts (group meetings and/or individual contacts), duration of interventional contacts and the combination with other strategies (construction of latrines and hand-washing mechanisms, micronutrient supplementation or food fortification and/or annual or biannual deworming etc.)^6,13,14,19–31^. Considering that those methodologic variabilities may in turn influence the outcomes of interventions, studies investigating if a nutrition-sensitive approach can reduce anemia better than a nutrition-specific intervention must standardize their design to allow comparability.

Previously we have designed a cluster-randomized controlled trial protocol aiming at determining the efficacy of educational community-based interventions in the reduction of anemia in preschool children, namely 1) Nutrition and 2) WASH/malaria education, both combined with a test-and-treat approach^32^. However, at the pre-intervention assessment the number of eligible clusters were found to be significantly lower than expected, the baseline characteristics of the participants were heterogeneous between the groups studied, and difficulties in assuring that interventions would be implemented under the ideal and highly controlled experimental conditions were anticipated. In the present study, we transitioned to an effectiveness cluster-randomized controlled trial. In doing so, small modifications were performed in the previous protocol, mainly in the main objective. Here, we primarily aim at testing whether a Nutrition and WASH/Malaria educational, community-based interventions were able to reduce anemia in pre-school children from Bengo (Angola), under real-world conditions. A beneficial point resulting from this design modification is the possibility of understanding if these interventions can produce effects under ecologically relevant conditions. On the other hand, lower internal validity and generalizability are expected to be associated to these alterations^33^. Three main hypothesis are being investigated, namely: (1) if adding a WASH/Malaria educational component to a test-and-treat therapeutic approach result in higher reduction of anemia, when compared to an exclusive therapeutic strategy, (2) if adding a nutrition educational component to a test-and-treat approach result in higher reduction of anemia, when compared to an exclusive therapeutic strategy, and 3) if the addition of a WASH/Malaria educational component (to a test-and-treat approach) result in higher reduction of anemia, than the addition of a nutrition educational component.

## Results

### Participant flow and characteristics

**Figure 1:**
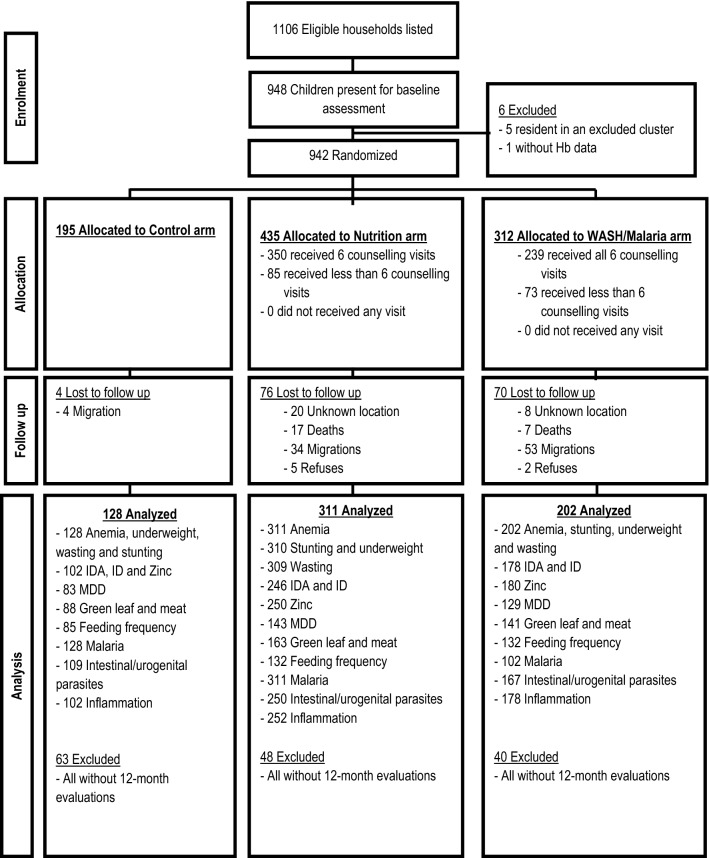
Children follow up flowchart. *Hb* hemoglobin, *IDA* iron deficiency anemia, *ID* iron deficiency, *MDD* minimum dietary diversity.

At the end of the census approach, 1106 households were considered eligible and the ones with the presence of the caretaker at the moment of the census were invited to participate. Of those, 830 caretakers and 948 children from seven hamlets attended the evaluation day at their local health centers. Furthermore, before randomization, one cluster was excluded due to a very low number of eligible resident children (only 5 children); despite that they continued to be followed. The number of participants randomly assigned to each arm and evaluated is described in Fig. 1.

Despite randomization, we found differences in the baseline characteristics between arms, regarding the children completing the study. For instance, the control arm had higher proportion of girls and children with *S. haematobium* infections, while children in the Nutrition arm had lower mean of hemoglobin levels and higher proportion of anemia, zinc deficiency, feeding frequency and green leaf consumption. Also, children in the WASH/Malaria arm had higher prevalence of Iron Deficiency and being infected with at least one intestinal/urogenital parasite (see Supplementary table S1). These variables were used to adjust the effectivity models for the heterogeneity in their distribution among study groups.

Dropouts occurred differentially between groups only regarding the mean of zinc levels (p-value = 0.042).

### Outcomes and estimation

Our results from crude difference-in-difference models showed no statistically significant differences between the 3 interventions, regarding the reduction of anemia and increasing of Hb (see Table 1). Nevertheless, differentiated variations between groups were found to occur in the prevalence of Iron Deficiency, Zinc deficiency and *P. falciparum* malaria (Wald chi-square p-value = 0.04, 0.04 and < 0.001, respectively). Those differences occurring specially between the Control and the Nutrition arms (Wald chi-square p-value = 0.077, p-value = 0.015 and p-value = 0.001, respectively).

**Table 1:** Effectiveness of interventions in reducing anemia and their associated factors.

Type of models	Crude models	Adjusted model 1				Adjusted model 2		
Comparations	Control vs. WASH/Malaria vs. Nutrition	Nutrition vs. Control		WASH/Malaria vs. Control		WASH/Malaria vs. Nutrition		WASH/Malaria vs. Nutrition Group with Interview score effect
Statistic evaluators	Wald chi-square p-value	% enhance	DDI (p-value)	% enhance	DDI (p-value)	% enhance	DDI (p-value)	DDI (p-value)
**Health primary outcome**								
Hb (Mean)	0.29	2.0	+ 0.151 (0.468)	0.7	0.023 (0.912)	− 1.3	− 0.160 (0.371)	0.023 (0.403)
Anemia (% yes)	0.22	− 17.2	− 0.424 (0.273)	0.3	0.263 (0.498)	22.8	+ 0.641 (0.050)	− 0.048 (0.332)
**Health secondary outcomes**								
IDA (% yes)	0.11	50.5	+ 0.468 (0.351)	24.2	+ 0.276 (0.606)	− 27.3	− 0.228 (0.594)	0.017 (0.776)
Iron deficiency (% yes)	**0.04**	71.6	** + 0.866 (0.032)**	− 5.3	+ 0.278 (0.498)	− 47.5	− 0.664 (0.144)	0.088 (0.258)
Zinc deficiency (% yes)	**0.04**	− 94.9	− **3.336 (0.006)**	− 103.7	− **2.126** (0.088)	169.5	+ 1.194 (0.117)	0.081 (0.411)
Weight (Mean)	0.88	0.6	+ 0.258 (0.291)	0.6	+ 0.281 (0.253)	0.0	− 0.029 (0.886)	− 0.006 (0.843)
Stunting (% yes)	0.29	23.3	+ 0.196 (0.610)	40.4	+ 0.217 (0.575)	11.8	0.043 (0.89)	− 0.041 (0.425)
Underweight (% yes)	0.55	–	–	–	–	–	–	–
Wasting (% yes)	0.44	–	–	–	–	–	–	–
*P. falciparum* (% yes)	** < 0.001**	− 317.7	− **3.052 (0.001)**	− 669.0	− 0.637 (0.433)	569.1	**2.495 (0.013)**	− 107 (0.351)
At least one intestinal/urogenital parasite (% yes)	0.79	− 129.1	− 0.073 (0.867)	− 6.9	− 0.316 (0.445)	49.8	0.244 (0.510)	− 0.061 (0.301)
Inflammation (% yes)	0.35	–	–	–	–	–	–	–
**Behavior Secondary outcome**								
Green leaf intake (% yes)	0.66	2,3	+ 0.261 (0.516)	15,9	+ 0.368 (0.385)	11.7	+ 0.103 (0.763)	0.009 (0.874)
Meat cons. (% yes)	0.72	− 12,0	− 0.230 (0.587)	− 10,6	− 0.262 (0.516)	− 1.1	− 0.058 (0.877)	0.048 (0.358)
Feeding frequency (Mean)		− 15,4	− 0.345 (0.164)	− 15,8	− 0.073 (0.769)	2.6	** + 0.416 (0.049)**	0.006 (0.864)
MDD (% yes)	0.73	–	–	–	–	–	–	–

Hb – Hemoglobin, IDA – Iron deficiency anemia. *difference-in-difference Wald chi-square, # Moderate-to-severe.

**Models 1**—Yit = β0 + β1 Time + β2 Group + δ (Time × Group) + β3 Gender + β4 Age + β5 Zinc Deficiency + β6 Feeding frequency + β7 Green leaf intake + β8 At least one intestinal/urogenital parasite + ε. **Model 2** (nutrition-WASH/Malaria) and **3** (nutrition-WASH/Malaria with interview scores)—Yit = β0 + β1 Time + β2 Group + δ (Time × Group) + β3 interview score + β4 Gender + β5 Age + β6 Zinc Deficiency + β7 Feeding frequency + β8 Green leaf intake + β9 At least one intestinal/urogenital parasite + ε.

Bold is highlighting statistically significant p-values.

When the regression models were adjusted, for the variables presenting differentiated prevalence profile at the baseline (and taking the Control arm as the reference), we found that the Nutrition group presented higher percentual enhancement in the prevalence of iron deficiency and lower enhancement of zinc deficiency and *P. falciparum* malaria prevalence (see Table 1). On the other hand, the WASH/Malaria group presented lower enhancement of zinc deficiency prevalence.

Furthermore, when we compared the adjusted WASH/Malaria educational effect to the Nutrition educational effect, we observed that children in the WASH/Malaria group had 22.8% higher prevalence of anemia than the Nutrition group. There were also higher prevalence of *P. falciparum* malaria and reported feeding frequency. We found no statistically significant associations between the variations in the outcomes and the number of domiciliary visits provided (see Table 1).

To further understand those results, link them to the interventions and assist with the interpretation, we performed additional ancillary analysis in each group (from the baseline to 12-month follow up) (described in Table 2).

**Table 2:** Variation in the primary and secondary outcomes among children completing the study, between the baseline and 12-month follow up.

Outcomes	Control				Nutrition				WASH/Malaria			
	T0	T2	Dif	P value	T0	T2	Dif	P value	T0	T2	Dif	P value
**Primary outcomes**												
Hb (Mean (SD))	11.2 (1.1)	11.3 (1.3)	0.05	0.706*	11.0 (1.4)	11.3 (1.3)	0.27	0.002*	11.3 (1.4)	11.4 (1.1)	0.13	0.210*
Anemia (% yes)	39.1 (50/128)	33.6 (43/128)	− 5.50	0.392**	50.5 (157/311)	36.3 (113/311)	− 14.2	< 0.001**	38.6 (78/202)	33.2 (67/202)	− 5.4	0.215**
**Secondary outcomes – Nutritional state**												
IDA (% yes)	24.5 (25/102)	10.8 (11/102)	− 13.73	0.016**	22.4 (55/246)	19.9 (49/246)	− 2.4	0.539**	21.9 (39/178)	13.5 (24/178)	− 8.4	0.032**
Iron deficiency (% yes)	12.7 (13/102)	7.8 (8/102)	− 4.90	0.359**	14.2 (35/246)	19.5 (48/246)	5.3	0.105**	24.2 (43/178)	18 (32/178)	− 6.2	0.185**
Zinc deficiency (% yes)	2 (2/102)	4.9 (5/102)	2.94	0.453**	12.8 (32/250)	3.6 (9/250)	− 9.2	< 0.001**	4.4 (8/180)	2.8 (5/180)	− 1.7	0.581**
Weight gain (Mean (SD))	9.2 (2.4)	12.1 (2.3)	2.84	< 0.001*	9.0 (2.5)	11.9 (2.1)	2.9	< 0.001*	9.1 (2.3)	12.0 (2.1)	2.9	< 0.001*
Stunting (% yes) #	29.7 (38/128)	37.5 (48/128)	7.81	0.143**	27.4 (85/310)	41.6 (129/310)	14.2	< 0.001**	22.3 (45/202)	39.1 (79/202)	16.8	< 0.001**
Underweight (% yes) #	20.3 (26/128)	18 (23/128)	− 2.30	0.678**	18.1 (56/310)	15.8 (49/310)	− 2.3	0.382**	21.3 (43/202)	14.9 (30/202)	− 6.4	0.066**
Wasting (% yes) #	9.4 (12/128)	6.3 (8/128)	− 3.10	0.454**	7.1 (22/309)	2.3 (7/309)	− 4.8	0.003**	10.9 (22/202)	5.4 (11/202)	− 5.5	0.052**
**Secondary outcomes – infections**												
*P. falciparum* (% yes)	6.3 (8/128)	21.1 (27/128)	14.85	< 0.001**	5.8 (18/311)	2.3 (7/311)	− 3.5	0.043**	1.5 (3/202)	6.4 (13/202)	4.9	0.021**
At least one intestinal/urogenital parasite (% yes)	16.5 (18/109)	48.6 (53/109)	32.11	< 0.001**	10 (25/250)	29.2 (73/250)	19.2	< 0.001**	22.8 (38/167)	53.3 (89/167)	30.5	< 0.001**
Inflammation (% yes)	51 (52/102)	41.2 (42/102)	− 9.80	0.174**	39.3 (99/252)	40.5 (102/252)	1.2	0.850**	48.9 (87/178)	44.9 (80/178)	− 3.9	0.494**
**Secondary outcomes – Feeding practices**												
Green leaf intake (% yes)	12.5 (11/88)	29.5 (26/88)	17.05	0.014**	31.9 (52/163)	49.7 (81/163)	17.8	0.002**	17.7 (25/141)	37.6 (53/141)	19.9	< 0.001**
Meat consumption. (% yes)	39.8 (35/88)	27.3 (24/88)	− 12.50	0.117**	33.7 (55/163)	17.2 (28/163)	− 16.6	< 0.001**	42.6 (60/141)	25.5 (36/141)	− 17.0	0.003**
Feeding frequency (> = 3)	57.6 (49/85)	75.3 (64/85)	17.65	0.014**	70.5 (93/132)	77.3 (102/132)	6.8	0.243**	59.1 (78/132)	67.4 (89/132)	8.3	0.177**
MDD (% yes)	13.3 (11/83)	16.9 (14/83)	3.61	0.664**	9.1 (13/143)	7.7 (11/143)	− 1.4	0.824**	17.1 (22/129)	17.1 (22/129)	0.0	1.000**

SD – Standard deviation, Hb – Hemoglobin, MDD – Minimum Dietary Diversity * Students’ t, ** McNemar Test.

Bold is highlighting statistically significant P values.

### Ancillary analysis

#### Comparing pre-and-post intervention moments (baseline to 12-month follow up variations)

In this study, an increased hemoglobin level (0.05 g/dl, 0.27 g/dl and 0.13 g/dl, respectively for the Control, Nutrition and WASH/Malaria arms) and a decreased prevalence of anemia (5.5%, 14.2% and 5.4% lower) were observed in all study arms, between the baseline and the 12-month follow up (Table 2). However, significant increased hemoglobin level and decreased anemia prevalence were only observed in the Nutrition arm.

Iron Deficiency Anemia (IDA) prevalence was found to be significantly reduced, either among the children in the Control (from 24.5% to 10.8%) and WASH/Malaria arms (from 21.9% to 13.5%), while no significant reduction on iron deficiency prevalence was observed (among the children with hemoglobin level ≤ 11 g/dl). The reduction of zinc deficiency prevalence was significant in the Nutrition arm, decreasing from 12.8% to 3.6%, from the beginning to the end of the study (see Table 2). Furthermore, despite that decreased wasting was observed in the Nutrition arm, the prevalence of stunting was found to increase 14.2% among the children in this group. Stunting among the children in the WASH/Malaria group also increased (16.8%).

Regarding infections, a significant variation in the prevalence of *P. falciparum* malaria was observed in all arms (from 6.3% to 21.1% in the Control arm, from 5.8% to 2.3% in the Nutrition arm and from 1.5% to 6.4% in the WASH/Malaria arm). Furthermore, the prevalence of children infected with at least one intestinal/urogenital parasite was also found to increase in all arms (32.1% in the control, 19.2% in the Nutrition and 30.5% in the WASH/Malaria arm) (see Table 2).

#### Comparing the 1st and 6th visits for behaviour change

More than 70% of the participants within the educational interventions successfully received 5-to-6 visits (70.8% of the households in the WASH/Malaria group and 71.8% in the Nutrition group). Furthermore, statistically significant increase in the proportion of households with latrine, clean latrines, clean environment and observed clean nails of the caretaker were observed in the WASH/Malaria group and significant increased weekly consumption of cereals, food from animal sources, legumes, vegetables, fruits and in the minimum feeding frequency was also observed in the Nutrition group (supplementary table S2).

## Discussion

Results from our crude and adjusted difference-in-difference models suggest that implementing two semesterly rounds of an exclusive test-and-treat therapeutic approach have no statistical significative difference in reducing anemia and increasing hemoglobin, that when combining this approach with 6 monthly domiciliary counselling visits addressing adequate child nutrition or WASH/Malaria practices. Considering the positive results reported by others, and the fact that our process indicators suggest the occurrence of some degree of behavior change in both educational intervention groups (supplementary table S2), these effectiveness results could suggest that, 1) the therapeutic component of the intervention played in fact the major role and educating had a residual effect, or 2) not all educational actions have been translated into changed behavior and in consequence the needed sequence of beneficial results happened incompletely (see supplementary Figure F1 and F2) and/or 3) the educational actions lacked the required intensity (number of monthly visits and duration of each session) and duration of the educational actions (only 12-months) to translate into significant changes at the primary outcomes^34,35^.

In fact, the way educational interventions are expected to impact in the primary outcomes constitute a long and complex pathway of actions, that may be subject to effect modifications among different populations. For instance, the therapeutic component (implemented in all study arms) was expected to clear infections that could be causing anemia; further allowing educational interventions to either improve WASH/Malaria practices that could reduce the infection transmission and allow the natural recovery of hemoglobin (in the WASH/Malaria arm); or to improve feeding practices that could lead to higher quantity and quality of diet and improve the hemoglobin status of children in the absence of infections (in the Nutrition arm). Nevertheless, at the end, all tree interventions were expected to increase hemoglobin levels and reduce anemia prevalence, besides improving other nutritional outcomes between the pre- and post-evaluation moments. Thus. plausibility arguments are recommended to be used as evidence of impact when evaluating the effect of real-world complex interventions^36^. Accordingly, we have analysed our data by protocol, rather than by intention to treat, to allow combining different types of evidences able to assist the interpretation of our results and to provide a more comprehensive discussion following the assumption mentioned above^37^.

For instance, instead of observing reduced prevalence of infections over a 12-month period (between the baseline and the 12-month follow), we observed a significant increase in the prevalence of having at least one intestinal/urogenital parasite (mainly *G. lamblia*, *S. haematobium* and *A. lumbricoides*) in all arms, and in the prevalence of *P. falciparum* malaria among the children within the Control and WASH/Malaria arms, contrary to other studies^4,6,25,29,38,39^. Assuming that baseline infections were cleared by the test-and-treat approach, this may associate with the occurrence of frequent/repetitive contact of children with highly contaminated environments, in turn influenced by the children age (as the wider mobility of older children may increasingly expose them to sources of contamination), which the WASH educational component wasn’t able to prevent^16,40^. That is corroborated by the similar longitudinal infectious profile of both Control and WASH/Malaria arms, and by the absence of significative differences in those variations in regression models. In the literature, while some studies report that adding chemotherapy to health education and sanitation can reduce the prevalence, intensity and/or incidence of *A. lumbricoides*, *T. trichiura*, hookworms or schistosomiasis and improve the knowledge, attitude and preventive practices toward those infections, others report no significant effect^6,25,26,29^. Similarly, malaria, educational packages were mentioned to improve the knowledge and preventive practices and treatment-seeking behavior, while integrating community case management and the possession of nets were reported to lower the prevalence and incidence of fever in under-five years old children^8,27,28,41^.

Also, adjusted models showed that the Nutrition group had significantly less *P. falciparum* prevalence than the Control group. This, could be related to seasonal malaria and ecological conditions^35,42^. Interestingly, the Nutrition group had also less zinc deficiency and more iron deficiency (consistent in crude models). To note that zinc is a trace element associated to the nutrition-dependent immune response (also called as nutritional immunity), being confined into cellular compartments during inflammatory events to decrease its concentrations and bioavailability to the pathogen^43–45^. Also, zinc levels can affect both tissue and/or systemic level responses to iron deficiency (affecting local iron regulatory proteins and/or the normal function of an iron regulatory hormone called hepcidin)^44,46–53^. Thus, we speculate that the reduction of *P. falciparum* malaria and zinc deficiency prevalence may have influenced each other, in turn affecting the prevalence of iron deficiency. Nevertheless, our results don’t allow to determine to which extent the educational approaches could have contributed differently to this fact.

Regarding the effect of educating for adequate IYCF practices, despite that a significant increase in the prevalence of reported consumption of green leaf in the previous 24 h (18.2%) was documented between the baseline and the 12-month follow up, children at the Nutrition group were reported to have consumed significantly less meat (− 16.9%) and lower feeding frequency (− 0.26) at the end of the study, when compared to the baseline. Additionally, the intervention failed at increasing the prevalence of Minimum Dietary Diversity (MDD) observed at the baseline. Those facts suggest that the educational component of the intervention may have had positive impact in some behavioral secondary outcomes but not in all of them (assumptions supported by our process indicators described in supplementary Table S2). Others reported that educational programs in nutrition may lead to children being fed more green leafy vegetables, nutrient-dense thick foods at lunch and meeting the dietary requirements for energy, iron and zinc more frequently and improved ability of mothers to identify malnutrition, despite that methodological variations may lead to different results^11,13,20,21^.

Two main operational limitations must be considered when interpreting our results. First, relates to the limitations documented during the implementation of the Test-and-Treat approach (performed in all study groups). Here, limitations in the implementation of the active screening and treatment of infections in the community was associated to failure to obtain samples (or to obtain the needed volume), samples collected in inappropriate containers (which may have introduced contaminations and misestimated frequencies), impossibility to perform Kato Katz in diarrheal samples (limiting the diagnosis of helminths), diagnosis made from single samples, failure of caretakers/children’s to attend treatment visits or consultations, non-supervision of all therapeutic dosage intake, difficulty in relocating households, and inadequate filling of treatment records by technicians (making the success of some treatments being inconclusive). Secondly, providing bed nets, soap and bleach to all groups may have encouraged caretakers to use it, potentially confounding the results of the Control and Nutrition arms. There was variation in the infectious profile of each cluster and that should also be taken into consideration.

It is reported that when moving from efficacy to effectiveness studies, we may also move to lower degree of generalizability^33,36^. Here, considering that randomization did not produced absolutely comparable groups, as differences regarding some baseline characteristics were observed between groups (i.e. the prevalence of some outcomes was not diluted within the clusters), generalizability is limited^54^. Thus, we recommend that the interpretation of results should be restricted to our settings and study populations. Furthermore, to use the difference-in-difference analysis in groups differing significantly at the baseline may wrongly suggest that changes occurred in one group and not in the other^55^. To minimize the impact of that issue in our results, we have adjusted the difference-in-difference models, accounting for variables whose baseline prevalence or value was statistically different between the groups being compared, as performed by others in similar studies (see supplementary Table S1)^56^. When interpreting our results, the reader must also consider that we have used a fixed number of clusters (of fixed size), and that it is reported that when the results of cluster randomized trials (with less than 40 clusters), are analysed using generalised equations there is an increased risk of inflated type I error (i.e., false positive findings)^55^. Nevertheless, some design aspects, commonly associated with efficacy research studies, were sustained in the effectiveness study implemented here, such as standardized design, structured interventions, focus on specific primary outcomes (while the improvement in behavior was kept as a secondary outcomes) and randomized assignment to interventions were still being implemented. Thus, our study may represent a hybrid between efficacy and effectivity, potentially allowing for a more systematized (real-life intervention) results, comparatively to traditional effectiveness studies.

## Methods

All methods in this study were carried in accordance to relevant guidelines and regulations, following both national regulatory norms and standards, as also international recommendations.

### Trial design

This study is a open (unblinded), parallel, cluster-randomized controlled trial, with 3 arms.

### Participants

#### Eligibility criteria

Children younger than 3 years old, resident in the selected hamlets, who were evaluated at the pre-intervention assessment, were considered eligible.

#### Recruitment, follow up and counselling visits

The initial assessment of participants occurred between March and May of 2015 (baseline that included 948 children), while the follow-ups occurred between November and December 2015 (6-month follow up that included 524 children) and between July and August of 2016 (12-month follow up that included 660 children). The counselling moments have intercalated those evaluation moments in 2 rounds of 3 counselling visits. The first occurred between June and October 2015 (included 610, 576 and 574 of the caretakers) and the second round occurred between January and June of 2016 (included 561, 574 and 563 of the caretakers).

### Settings and locations where the data were collected

The data was collected in 7 administratively and geographically isolated hamlets with health facilities, located within the CISA Health and Demographic Surveillance System (HDSS) study area, and that were found to provide daily primary care at the pre-intervention assessment. The DHSS area comprehend essentially the communes of Úcua, Caxito and Mabubas, in turn located within the Dande municipality of the Bengo province (in Angola). Detailed characterization of demography, socioeconomic aspects, causes of death and epidemiology of some diseases have already been published^57–65^.

### Interventions

#### Therapeutic (test-and-treat) component

This component was included in all study arms and was conducted and implemented at the evaluation moments. Briefly, *P. falciparum* infections were screened and treated at the evaluation sites, while urogenital schistosomiasis and/or intestinal parasites were diagnosed at the CISA´s laboratory and treated at specific domiciliary visits (in the case of treatments with albendazole) and/or hospital-based consultations (in the case of treatments with praziquantel)^32^. A standard treatment protocol, following the national therapeutic guidelines and specific bibliography, was used. Furthermore, children diagnosed with sickle cell disease were referred to the Anemia Patient Follow-up Consultation, held at the Bengo General Hospital.

#### Educational component

As previously published, caretakers of children allocated to the educational interventions in Nutrition or WASH/Malaria have received six domiciliary, personalized counselling sessions^32^. Those educational visits were conducted from June of 2015 and June of 2016), having 3 evaluation moments being implemented before they start (at the baseline), after 3 of those visits (first follow up) and after the sixth visit (second follow up). Briefly, counselling to the educational intervention groups targeted eight main topics. Regarding the nutrition group, the main topics were breastfeeding, complementary feeding, weekly adequate food diversity, appropriate number of meals, adequate amount of food, responsible feeding, food and disease, and food safety and hygiene. On the other hand, the WASH/Malaria group was counselled on insecticide-treated nets (ITNs) usage, reducing mosquito breeding sites, prevention of open sky defecation, latrine cleaning, adequate hand washing, healthy backyard environment, adequate water availability, treatment, transportation and storage and adequate personal hygiene as previously described^32^.

### Socio-economic and demographic data and biological specimen collection and laboratory testing

The sample collection and processing have been previously described in detail^32^. Briefly, a standardized questionnaire was administered by trained interviewers to mothers or caretakers, aiming at collecting socio-economic and demographic information, as well as information regarding pregnancy and breastfeeding, complementary feeding practices, health care, food security, WASH and malaria practices^66,67^. Weight, height and mid-arm circumference were measured and used to calculate anthropometric indices for the diagnosis of undernutrition, according to WHO standards^68^. Blood was collected by venous puncture according to WHO recommendations for neonates and young children and used for 1) the diagnosis of *P. falciparum,* through Rapid Diagnostic Tests (SD bioline Malaria Ag P.f*/*P.v, Standard Diagnostics, Inc., Republic of Korea) and 2) biochemical analysis for the quantification of blood levels of hemoglobin (using an Hemocue Hb 301 System, Angelholm, Sweden) and serum levels of ferritin, Protein-C- Reactive and Zinc (using an automated autoanalizer (BT1500) from Biotecnica Instruments S.p.A, Rome, Italy) and CRP turbidimetric latex, Ferritin and Zinc kits from Quimica Clínica Aplicada S.A., Tarragona, Spain)^69^. The diagnosis of intestinal parasites in stool samples was performed using Kato-Katz technique and Parasitrap kits (Biosepar, Germany) and urogenital schistosomiasis was diagnosed by urine filtration, using Whatman Nuclepore membranes (diam. 25 mm, pore size 12 μm, polycarbonate, Merck, Germany)^70–72^.

### Outcomes

Primary outcomes comprised only health indicators, namely increase in hemoglobin levels and reduction of anemia prevalence, as described by Fançony et al. 2019^32^. Secondary outcomes comprised health and behavior indicators for the estimation of impact evaluations, while process indicators (which comprised either reported or observed practices or behaviors collected at the counselling visits) were used to conduct process evaluations. The complete list of outcomes and their analysis strategy within each arm is presented Table 3.

**Table 3:** Outcomes and analysis performed in this study.

Variables	WASH/Malaria	Nutrition	Control
**1) Primary outcome:** **1.1)** Impact on health measured at the evaluation moments*			
Hemoglobin levels (g/dl)	x	x	x
Anemia prevalence (%)	x	x	x
**2) Secondary outcomes:** 2.1) Impact on health measured at the evaluation moments*			
Iron deficiency anemia (%)	x	x	x
Weight (kg)	x	x	x
Stunting (%)	x	x	x
Underweight (%)	x	x	x
Having at least one intestinal/urogenital parasite (%)	x	x	x
*P. falciparum* (%)	x	x	x
2.2) Impact on behaviour reported at evaluation moments*			
Minimum dietary diversity (MDD) (%)	x	x	x
Green leaf consumption (in previous 24 h) (%)	x	x	x
Meat consumption (in previous 24 h) (%)	x	x	x
Children sleeping under bednets in the previous night (%)	x	x	x
2.3) Impact on behaviour observed by the technician at the WASH/Malaria educational sessions**			
Nº of domiciliary counselling visits delivered (%)	x	NA	NA
Having bednet in the children’s bed (%)	x	NA	NA
Latrine ownership (%)	x	NA	NA
Having water to wash hands in the latrine (%)	x	NA	NA
Clean latrine (%)	x	NA	NA
Backyard environment with garbage (%)	x	NA	NA
Backyard environment with loose animals (%)	x	NA	NA
Backyard environment with still water (%)	x	NA	NA
Caretaker having clean nails (%)	x	NA	NA
2.4) Impact on behaviour: reported by caretakers at the nutrition educational sessions**			
Nº of domiciliary counselling visits delivered (%)	NA	x	NA
Weekly cereal consumption (%)	NA	x	NA
Weekly seeds consumption (%)	NA	x	NA
Weekly Milk and derivatives consumption (%)	NA	x	NA
Weekly Food from animal sources consumption (%)	NA	x	NA
Weekly Eggs consumption (%)	NA	x	NA
Weekly Legumes consumption (%)	NA	x	NA
Weekly Vegetables consumption (%)	NA	x	NA
Weekly Fruits consumption (%)	NA	x	NA
Feeding frequency (%)	NA	x	NA

x Evaluated; *Mean and frequency variation between baseline and the 12-month follow up; ** frequency variation between the first and the sixth counselling visits; NA – Non applicable.

### Sampling strategy

The sampling strategy chosen for this study was a non-probabilistic (convenience) sampling^32^. Eligible children were listed and invited to participate using a census approach, within a fixed number of clusters. This strategy was adopted because variations in the density of eligible children were expected and the real density in each cluster was uncertain.

### Randomization

For this study we used a block randomization, where hamlets (considered as cluster units) were randomly allocated to each study arm. At the end of the pre-evaluation assessment, only 6 hamlets were found to be eligible and were randomized to the study arms. For the randomization, the names of the hamlets were written down on pieces of paper, placed in a bag and successively removed. The first two papers removed were attributed to the Nutrition arm, the following two to the WASH/Malaria arm and the next pair to the control arm. This process was carried out by the study researchers.

### Statistical analysis

SPSS software (version 25.0, International Business Machines Corporation, Pittsburgh, PA) was used for statistical analyses.

Prevalence was calculated as the frequencies of the outcome over the total number of samples with valid results and prevalence reduction (PR) was calculated as the difference between the proportion of prevalence at the baseline *vs* 12-month after the beginning of the study.

To investigate the differences in the baseline characteristics, between the children completing the study and the ones dropping out after 12-months, we used Chi-square Test for the categoric variables and T Student for the continuous variables. Logistic regression models were used to analyze interactions between the study arms and variables (using dropouts as dependent variables) and determine if losses were differentiated between arms. Thus, the existence of differences in the baseline characteristics between study arms (for children completing the study) was investigated using ANOVA and Chi-square, respectively to continuous and categoric variables.

Students’ t and McNemar tests were used to determine longitudinal variations on the primary and secondary outcomes between baseline and the end of the study (respectively at the pre and post-interventional moments) within each arm.

To determine the differences in the crude variations between the three study arms, the difference-in-difference technique was performed using Fit Generalized estimating models. In summary, linear regression models were used to estimate the mean hemoglobin level and weight variations and logistic regression models were used to estimate the variation on the prevalence of anemia and other secondary binary outcomes. In each model, independent variables were time (0 = T0 and 1 = T1), the group, and group*time interaction. Outcomes presenting significant longitudinal variations within each arm, or significant crude differences when comparing study arms, were selected to be further inspected for significant differences in adjusted models. Adjustment was conducted by including secondary outcomes whose prevalence was found to be differentially distributed among the three arms at the baseline (considered as potentially confounding variables) and also age (found to influence the occurrence of anemia in previous studies). The interaction term (represented by DDI) indicates whether there were statistically significant differences in the adjusted variation of T0 to T1 between groups, as previously described by Mahfuz et al.^56^. The Control group was used as the reference group when determining the effectiveness of the therapeutic-plus-educational intervention, comparatively to an exclusive therapeutic (test-and-treat) intervention, while the Nutrition group was used as the reference group when determining the effectiveness of the WASH/Malaria educational intervention (further accounting for the number of interviews conducted in both educational interventions). The quantification of the crude percent of enhancement in the haemoglobin level and in the prevalence of anemia and associated factors was calculated as described by Mahfuz et al.^56^.

Process indicators were collected at every counselling visits (from a total of six visits) conducted in Nutrition and WASH/Malaria arms. Changes in the proportion of adequate behaviour or practices were calculated between the first and the sixth visits, using McNemar tests. Adequate household behavior/practices in the WASH/Malaria arm included observed bednet in the children’s bedroom, clean latrine, latrine with water to wash hands (either with current or still water mechanisms), clean backyard environment, namely without garbage, loose animals, still waters and caretaker with clean nails. In the Nutrition group, adequate behavior/practices included the reported weekly consumption of cereals, seeds, milk and milk derivatives, food of animal origin, eggs, legumes, vegetables, other fruits and the minimum feeding frequency. “Sustained behavior/practice” was considered to be a behavior or practice observed in both first and sixth visits and “Changed behavior” was considered to be an altered behavior or practice between the first and sixth visits.

### Ethical considerations

After the explanation of the study, an informative brochure was given to the caretakers. This informative document was illustrative to allow for easy comprehension. Illiterate caretakers were encouraged to additionally ask for a carefully reading the content of the document at home by a literate family member and to further ask for additional information at any point of the study. Thereafter, they were asked to sign an informed consent in order to formalize their acceptance and commitment in their children participation. The collected data were computerized and archived to ensure the confidentiality and privacy. Children with positive for malaria, urogenital schistosomiasis and intestinal parasites diagnostic tests were treated with Artemether-Lumefantrine, albendazole (ALB) and Praziquantel (PZQ) at the baseline, and 6 and 12 months follow ups (according to a therapeutic protocol approved by a pediatrician and the director of Bengo General Hospital). Follow up consultations were scheduled for children with severe undernutrition at the malnutrition department at the Bengo General Hospital and children with severe anemia were forwarded to the pediatric emergency department of that hospital. Mothers not taking their children to those consultations were visited to understand the reasons for that and to explain to them the importance of their compliance. This study was approved by the Angolan Ministry of Health Ethics Committee.

### Harms

No harms have been documented during the implementation of this study.

### Registration

This study is registered with the title: Efficacy of community educational interventions in nutrition and WASH/Malaria in reducing anemia in under 5 children, in the municipality of Dande – Angola, at www.isrctn.com (https://doi.org/10.1186/ISRCTN18101157), with the trial identifier number ISRCTN: 18101157. The date of registration is 06/09/2016.

### Protocol

The original protocol can be assessed at https://www.ncbi.nlm.nih.gov/pubmed/30764549.

## Conclusions

In this study, adding a 12-month educational Nutrition or a WASH/Malaria component to a test-and-treat approach had no greater beneficial effect reducing the prevalence of anemia and increasing the level of hemoglobin than an exclusive therapeutic approach. Possibly, the intensity (number of counselling visits and length of each session) and duration of the educational actions may haven’t resulted in the amount of behavior change needed to stop transmission or improve the general child feeding practices, as reported by others, and should be addressed in future studies. Nevertheless, this is the first study published in Angola simultaneously testing the effect of three main strategies to prevent and reduce anemia. Besides comparing the performance of these interventions in reducing anemia in this context, which allows for evidence-based planification of future targets, this study also provides detailed data on how the frequency of immediate determinants varies under each intervention during a 12-month follow up. Considering that anemia and undernutrition had similar proximal determinants, the application of these results may have broader public health application.

## Supplementary Information

Supplementary Information.
