# Supplementary material for: Effectiveness of Nutrition and WASH/malaria educational community-based interventions in reducing anemia in children from Angola
Source: Sci Rep. 2021 Mar 10;11:5603. doi: 10.1038/s41598-021-85006-x (PMC7946872; doi:10.1038/s41598-021-85006-x)
Supplement: Supplementary file 1 — Supplementary Information. [file 41598_2021_85006_MOESM1_ESM.docx]

**Effectiveness of nutrition and WASH/malaria educational community-based interventions in reducing anemia in children from Angola: a cluster-randomized controlled trial**

Cláudia Fançony^1, 2^ *, Ânia Soares^1^, João Lavinha^3,5^, Henrique Barros^2#^ and Miguel Brito^1,4#^

*Supplementary table S1 – Between-group-differences regarding the prevalence and mean distribution of variables at the baseline*

|  | **Control (N=195)** | **Nutrition (N=435)** | **WASH/Malaria (=312)** | **p-value** |
| --- | --- | --- | --- | --- |
| Age – M (SD) | 17.3 (10.3) | 16.5 (10.3) | 16.9 (9.6) | 0.575* |
| Gender: Girls | 55.4% (108/195) | 42.1% (183/435) | 52.9% (165/312) | **0.001**** |
| Hb – M (SD) | 11.2 (1.3) | 11.0 (1.4) | 11.3 (1.3) | **0.015*** |
| Anemia: yes | 39.0% (76/195) | 52.2% (227/435) | 37.5% (117/312 | **<0.001**** |
| IDA: yes | 20.2% (33/163) | 23.1% (88/381) | 19.4% (55/283) | 0.489** |
| Weight– M (SD) | 9.0 (2.5) | 8.9 (2.5) | 8.9 (2.2) | 0.861* |
| Stunting (moderate-to-severe): yes | 28.5% (55/193) | 27.8% (120/432) | 24.7% (77/312) | 0.548** |
| Underweight (moderate-to-severe): yes | 20.2% (39/193) | 18.8% (81/432) | 22.1% (69/312) | 0.529** |
| Wasting (moderate-to-severe): yes | 10.4% (20/193) | 8.8% (38/431) | 10.3% (32/312) | 0.745** |
| Iron deficiency: yes | 24.0% (23/96) | 27.3% (47/172) | 40.2% (70/174) | **0.007**** |
| Zinc deficiency: yes | 1.8% (3/164) | 11.2% (44/392) | 3.8% (11/290) | **<0.001**** |
| Inflammation: yes | 48.5% (79/163) | 42.0% (165/393) | 48.1% (139/289) | 0.191** |
| Feeding frequency: M (SD) | 2.9 (1.7) | 3.2 (1.7) | 2.9 (1.5) | **0.018*** |
| MDD: yes | 15.7% (25/159) | 14.5% (43/297) | 18.0% (45/250) | 0.531** |
| Green leaf intake: yes | 16.6% (28/169) | 30.3% (103/340) | 17.3% (48/277) | **< 0.001**** |
| Meat consumption: yes | 37.9% (64/169) | 33.8% (115/340) | 43.3% (120/277) | 0.054** |
| Malaria: yes | 6.7% (13/195) | 6.2% (27/435) | 2.9% (9/311) | 0.078** |
| Having at least one intestinal/urogenital parasite: yes | 19.1% (34/178) | 10.6% (41/386) | 19.7% (52/264) | **0.002******** |

M (SD) – Mean and standard deviation, Hb – Hemoglobin, MDD – Minimum Dietary Diversity. *ANOVA; **Chi-square Test. P-values in bold are significant at the 95% level.

*Supplementary table S2– Results from comparing the first and sixth visits for behaviour change*

In the WASH/Malaria arm, 70.8% of the households successfully received 5-to-6 visits (out of a maximum of six). Furthermore, statistically significant increase in the proportion of households with latrine (5%, p-value=0.043), clean latrines (26.7%, p-value<.001, clean environment (23.0%, p-value<0.001) and observed clean nails of the caretaker (24.8%, p-value<0.001) were observed, while significant decrease in the proportion of households having garbage (-14.2%, p-value<0.001), loose animals (27.2%, p-value<0.001) and/or still waters (-14.0%, p-value<0.001) in their backyard were documented. Between the initial and final educational interviews, 26.4% (56/212) of the households that initially had a bednet in the children’s bedroom sustained its use in the last visit, while 12.7% (27/212) did not. Furthermore, 41.0% (87/212) of the households that were observed not to be using the bednet to protect the children remain not using them at the end of intervention despite being advised to do so, whereas 19.8% (42/212) where observed to have changed the inadequate behavior. Besides, changes from inadequate to adequate behavior/practices were observed in 29.0% (54/186), 9.7% (18/185), 25.4% (54/213), 19.2% (41/213), 26.8% (57/213), 17.1% (36/211) and 26.8% (56/209) of the households/caretakers for respectively, having a clean latrine, having water to wash hands in the latrine, having a clean backyard environment, not having garbage, loose animals or still water in the backyard and having clean nails.

Regarding the process indicators collected during the educational visits in the nutrition arm, we observed that 71.8% (312/435) of the caretakers have participated in 5 or 6 domiciliary counselling visits, while only 10.6% (46/435) did not participate in any interview. Also, significant increased weekly consumption of cereals (11.6%, p-value<0.001, food from animal sources (26.3%, p-value<0.001), legumes (31.9%, p-value<0.001), vegetables (16.6%, p-value<0.001), fruits (14.5%, p-value<0.001) and in the minimum feeding frequency (35.3%, p-value<0.001) was also observed (see the table bellow). Furthermore, 12.5% (34/272), 18.5% (50/271), 20.1% (54/268), 29.0% (78/269), 15.7% (42/268), 41.2% (110/267), 20.0% (54/270), 24.0% (65/271), 41.3% (114/276) of the children that initially were reported not to have consumed weekly cereals, seeds, milk and milk derivatives, food of animal origin, eggs, legumes, vegetables, other fruits and to not have met the minimum feeding frequency were classified as doing so at the end of the intervention.

Supplementary table S2– Variations in the process indicators between the first and last counselling visits

|  | First counselling visit | | | Sixth counselling visit | | |  |  |  |  |
| --- | --- | --- | --- | --- | --- | --- | --- | --- | --- | --- |
| Process indicators | % | n | N | % | n | N | Difference | Mcnemar p-value | N |  |
| Observed behaviour/practices at the WASH/Malaria group | | | | | | | | | | |
| Bednet usage (Yes) | 38.0 | 98 | 258 | 46.1 | 106 | 230 | 8.1 | 0.091 | 212 |  |
| Latrine ownership (Yes) | 58.3 | 151 | 259 | 63.3 | 145 | 229 | **5.0** | **0.043** | 212 |  |
| Clean latrine (Yes) | 64.5 | 147 | 228 | 91.2 | 196 | 215 | **26.7** | **<0.001** | 186 |  |
| Having water to wash hands in the latrine (Yes) | 10.5 | 24 | 228 | 15.4 | 33 | 214 | 4.9 | 0.265 | 185 |  |
| Having current water to wash hands in the latrine (Yes) | 3.1 | 8 | 256 | 2.7 | 6 | 225 | **-0.5** | 1.000 | 205 |  |
| Having still water to wash hands in the latrine (Yes) | 96.1 | 246 | 256 | 97.8 | 223 | 228 | **1.7** | 0.424 | 208 |  |
| Clean backyard environment (Yes) | 75.7 | 196 | 259 | 98.7 | 227 | 230 | **23.0** | **<0.001** | 213 |  |
| Backyard with garbage (Yes) | 47.3 | 122 | 258 | 33.0 | 76 | 230 | **-14.2** | **<0.001** | 213 |  |
| Backyard with loose animals (Yes) | 49,8 | 129 | 259 | 22,6 | 52 | 230 | **-27,2** | **<0.001** | 213 |  |
| Backyard with still water (Yes) | 17,5 | 45 | 257 | 3,5 | 8 | 230 | **-14,0** | **<0.001** | 211 |  |
| Caretaker having clean nails (Yes) | 70,0 | 177 | 253 | 94,8 | 218 | 230 | **24,8** | **<0.001** | 209 |  |
| Reported child feeding practices at the nutrition Group | | | | | | | | | | |
| Cereal consumption* (Yes) | 88,1 | 288 | 327 | 99,7 | 320 | 321 | **11,6** | **<0.001** | 272 |  |
| Seeds consumption* (Yes) | 61,9 | 203 | 328 | 58,2 | 185 | 318 | -3,7 | 0.163 | 271 |  |
| Milk and derivatives consumption * (Yes) | 39,1 | 127 | 325 | 32,0 | 102 | 319 | -7,1 | 0.239 | 268 |  |
| Food from animal sources consumption * (Yes) | 69,7 | 225 | 323 | 96,0 | 308 | 321 | **26,3** | **<0.001** | 269 |  |
| Egg consumption * (Yes) | 32,1 | 104 | 324 | 28,2 | 90 | 319 | -3,9 | 0.159 | 268 |  |
| Legume consumption * (Yes) | 55,9 | 180 | 322 | 87,8 | 280 | 319 | **31,9** | **<0.001** | 267 |  |
| Vegetable consumption * (Yes) | 80,3 | 261 | 325 | 96,9 | 311 | 321 | **16,6** | **<0.001** | 270 |  |
| Fruits consumption * (Yes) | 70,9 | 231 | 326 | 85,4 | 274 | 321 | **14,5** | **<0.001** | 271 |  |
| Minimum feeding frequency (Yes) | 22,0 | 72 | 327 | 57,3 | 185 | 323 | **35,3** | **<0.001** | 276 |  |

*Weekly consumption

Supplementary Figure F1 - Conceptual framework of the way WASH/Malaria educational intervention is expected to impact in the primary outcomes.

As deworming is expected to treat infections and automatically interrupting the processes by which infections lead to micronutrient deficiencies, decrease hemoglobin and cause anemia, the educational component of this intervention is expected to prevent the children from acquiring or reacquiring those infections (by limiting the occurrence of new cases and reinfections), based on the assumption that promoting adequate Water, Sanitation and Hygiene (WASH) practices and basic Malaria preventive practices would improve the knowledge and raise awareness of caretakers (regarding the causes, prevention and treatment of malaria, urogenital schistosomiasis and intestinal parasites), that caretakers would in turn improve mother-to-child practices and that those changes in behavior would be reflected in children being less exposed to the infective stages of intestinal and urogenital parasites and malaria (see next figure). As mentioned, limiting the impact of infections should allow that a normal diet and physiologic processes restore the nutritional status of children.

Supplementary Figure F2 - Conceptual framework of the way Nutrition educational intervention is expected to impact in the primary outcomes.

In this study group, while the test-and-treat approach is expected to clear infections that may cause anemia, the educational intervention in nutrition is expected to reduce nutritional anemia based on the assumption that 1) it would improve the knowledge and awareness of mothers and caretakers regarding adequate feeding practices for young children, 2) which in turn could be translated into better mother.-to-child feeding practices, 3) resulting in improved nutritional diversity and consumption of target foods (rich in micronutrients needed to erythropoiesis) by the child, which could finally be reflected into 4) increased hemoglobin levels and decreased anemia.
